# Integrative analysis of young genes, positively selected genes and lncRNAs in the development of *Drosophila melanogaster*

**DOI:** 10.1186/s12862-014-0241-9

**Published:** 2014-12-04

**Authors:** He-Qun Liu, Yan Li, David M Irwin, Ya-Ping Zhang, Dong-Dong Wu

**Affiliations:** State Key Laboratory of Genetic Resources and Evolution, Kunming Institute of Zoology, Chinese Academy of Sciences, Kunming, China; Kunming College of Life Science, University of Chinese Academy of Sciences, Kunming, Yunnan China; Department of Laboratory Medicine and Pathobiology, University of Toronto, Toronto, Canada; Banting and Best Diabetes Centre, University of Toronto, Toronto, Canada

## Abstract

**Background:**

Young genes and genes under positive selection commonly contribute to adaptive phenotypic evolution. Early developmental stages are very important for establishing phenotypes, which might be helpful for studying the evolutionary patterns of these rapidly evolving genes.

**Results:**

Here, we performed a weighted gene co-expression network analysis to identify modules of co-expressed genes at different stages of *Drosophila melanogaster* development. We found that young genes, including duplicated, orphan, and young lncRNA genes, are significantly enriched among modules associated with specific developmental stages. In addition, genes undergoing rapid amino acid sequence evolution driven by positive selection showed a similar proportion of essentiality with other genes, and enrichment in modules for specific developmental stages.

**Conclusions:**

Our integrative analysis revealed important roles for the origin of new genes and rapid amino acid sequence evolution in development that may account for specific phenotype evolution in *Drosophila melanogaster*.

**Electronic supplementary material:**

The online version of this article (doi:10.1186/s12862-014-0241-9) contains supplementary material, which is available to authorized users.

## Background

Evolutionary biologists have long sought to understand the molecular genetic basis of phenotypic evolution. Britten and Davidson proposed that regulatory mutations play a crucial role in phenotypic evolution from their observations that a substantial proportion of repetitive sequences regulate expression in genomes [1]. A pioneering study by King and Wilson found that the degree of divergence in protein sequences cannot account for the phenotypic differences between human and chimpanzee, and proposed that the evolution of anatomy was influenced to a greater extent by changes in gene regulation than by changes in protein sequence [2]. Recently, Carroll has put forward the proposal that the evolution of animal morphology is mainly attributable to mutations in the cis-regulatory regions of genes, instead of mutations in their protein-coding regions [3-6]. With the advent of large-scale genomic data, the role of positive selection in the evolution of protein coding sequences involved in phenotypic evolution is now known to have been underestimated. For example, the coding sequences of many genes involved in the development of the human brain have been shown to have evolved under positive selection, which may account for the rapid evolution of the human brain [7-13]. Adaptive changes in the amino acid sequence of MC1R account for pigment adaptation including variations in skin and hair color in many species [14]. Genome-wide surveys of polymorphism in humans have estimated that >10% of the amino acid substitutions in sequences that occurred between humans and chimpanzee may have been adaptive [15,16]. During short-term evolution within species, most mutations that cause morphological variation have been found in protein-coding regions [17]. In contrast, during the long-term evolution between species most mutations that cause morphological differences have been found in cis-regulatory regions. It has been proposed that this difference in abundance of coding and regulatory mutation in morphological characters is due to coding mutations within species not being able to spread through populations due to their pleiotropic deleterious effects [17].

New genes also provide crucial material for evolutionary innovations. Accumulating evidence supports the remarkable phenotypic effects of new genes in development [18-20]. For example, a pioneering study by Chen et al (2010) found that many new genes rapidly evolve to gain essential functions in *Drosophila*, since knockdown experiments of many of these new genes by RNA interference (RNAi) leads to lethality at various development stages. It was also found that some of these new genes have important functions in the process of organogenesis in *Drosophila* [21]. A functional association between new genes and newly evolved brain structure was observed in humans [22,23] and *Drosophila* [24]. In the human genome many new genes appear to have been recruited to function in the development of the brain [18,22,23,25].

Evolution of phenotypes is mainly determined by changes that occur in developmental processes. Examination of evolutionary changes occurring in genes expressed during development should be helpful for understanding the roles of genetic innovation in phenotypic evolution. Here, based on an analysis of gene co-expression networks at different developmental stages, we describe important roles for new genes, and rapid amino acid sequence evolution in genes, in development that might be responsible for specific aspects of phenotypic evolution in *Drosophila melanogaster*.

## Results

### Analysis of gene co-expression networks derived from transcriptomes of different developmental stages of *drosophila melanogaster*

Transcriptomes from early developmental stages and diverse adult tissues in *Drosophila melanogaster* studied in [26] were used for identifying new transcripts and calculating the expression values for genes (see Materials and Methods, Additional file 1). Genes involved in the same biological process tend to have expression levels that are highly correlated across samples. Identification of groups of co-regulated genes, or “modules”, should be informative for identifying specific features of developmental stages/tissues/cells. We implemented a weighted gene co-expression network analysis (WGCNA) [27] to identify modules of co-expressed genes during different stages of development in *Drosophila melanogaster*. This approach has been successfully employed to deduce regulatory networks in the developing human brain [28,29]. Here, a total of 46 coexpression modules (labeled numerically, e.g., M29, and by color, e.g., black, see Additional file 2) were obtained for *Drosophila* development. Many of the modules were developmental stage-specific (Additional file 3), for example, M2 is correlated, with high significance, with the 2-4 hr embryonic developmental state (P = 5e-42), and M39 is strongly correlated, with high significance, with adult females 30 days after eclosion (P = 2e-10).

**Figure 1:**
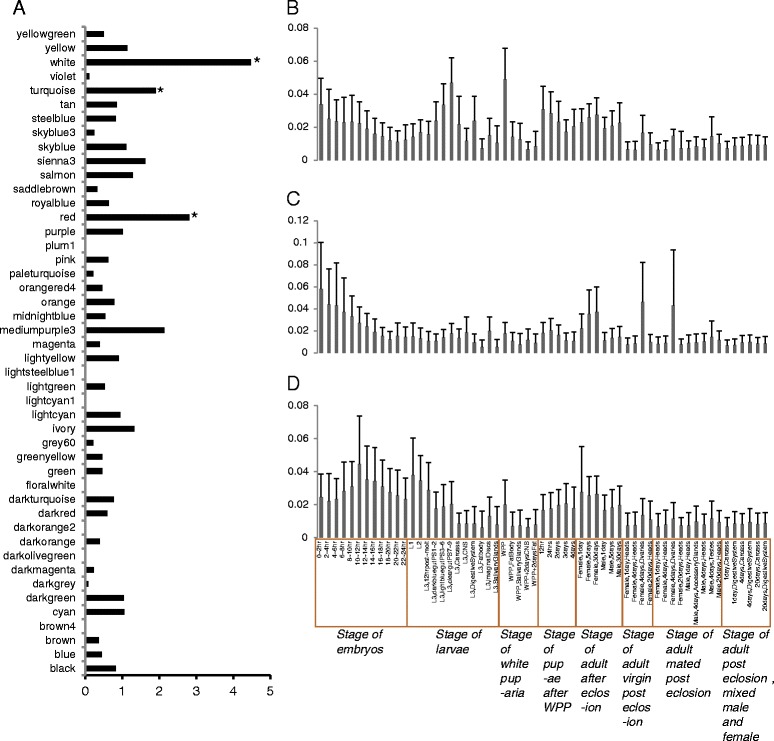
**Expression profile of completely lethal genes in each module. (A)** Enrichment of completely lethal genes in gene modules. Stars identify modules with significant enrichment for lethal genes. **(B-D)** Histogram of the expression of genes in modules M17 **(B)**, M6 **(C)** and M28 **(D)** and heatmaps of the expression of genes in this modules are seeing in Additional files 4 and 5. Expression of genes in each developmental stage was calculated as the expression in the stage divided by the sum of all stages. Mean expression and standard deviation (SD) of all genes in each stage are shown.

The Bristle Screen online database [30] was used to search for essential genes (here, a gene was deemed as essential for survival if it was completely lethal with constitutive RNAi). This database contained 20,262 transgenic RNAi lines that are predicted to target 11,619 of the 14,139 protein-coding genes (82.2%) in release 5.7 of the *Drosophila* genome. Lethality described in this database included several categories: completely lethal (no adults of the desired genotype, 1803 lines); some lethality (adult “escapers” had no obvious phenotype, 506 of lines); and lines that showed some phenotype with the accompanying lethality (1,230 lines, 1,069 genes). Of the 15,379 Drosophila reference genes present in our modules, 10,753 were included in the 18,663 lines with knock-down data in the Bristle Screen database, with 1,237 genes (in 1,655 lines) showing completely lethality (~8.868%). The analysis was then expanded to include all of the genes (i.e., not just reference Drosophila genes) represented in our modules. A total of 11,632 genes, represented by 20,262 RNAi lines, from the Bristle Screen database, were represented in our modules, with 1,339 of the genes (from 1,803 lines) showing complete lethality (~8.898%). From this analysis, three modules, M17, M6 and M28, were identified as being significantly enriched with lethal genes (P = 1.84E-22, P = 3.61E-62 and P = 1.03E-22, respectively, by the *χ*^2^ test, after FDR correction) (Figure 1A). Expression of genes in module M17 are significantly associated with the white prepupae (WPP) stages (P = 6e − 04, Figure 1B, Additional file 4), genes in module M6 are significantly correlated with the 0-2 hr embryo stage (P = 4e − 04, Figure 1C, Additional file 5) and genes in module M28 are significantly associated with the 10-12 hr embryo development stage (P = 0.01, Figure 1D, Additional file 6), revealing essential roles for these highly expressed genes in these developmental stages. The 0-2 hr embryo stage, i.e. the specific stage of module M6, is the maternal to zygotic transition period in *Drosophila* [31]. Consistent with this period in development, gene ontology analysis revealed that genes in module M6 are enriched in biological categories associated with chromatin organization (P = 2.52e-37, after FDR correction), transcription (P = 7.40E-32, after FDR correction), cell cycle (P = 1.07E-29, after FDR correction), development (P = 2.88e-04, after FDR correction) and morphogenesis (P = 8.79e-04, after FDR correction) (Additional file 7). These data suggest an essential function for these genes in the maternal to zygotic transition period.

### Developmental stage-specific enrichment of new genes revealed by gene co-expression networks in *Drosophila melanogaster*

**Figure 2:**
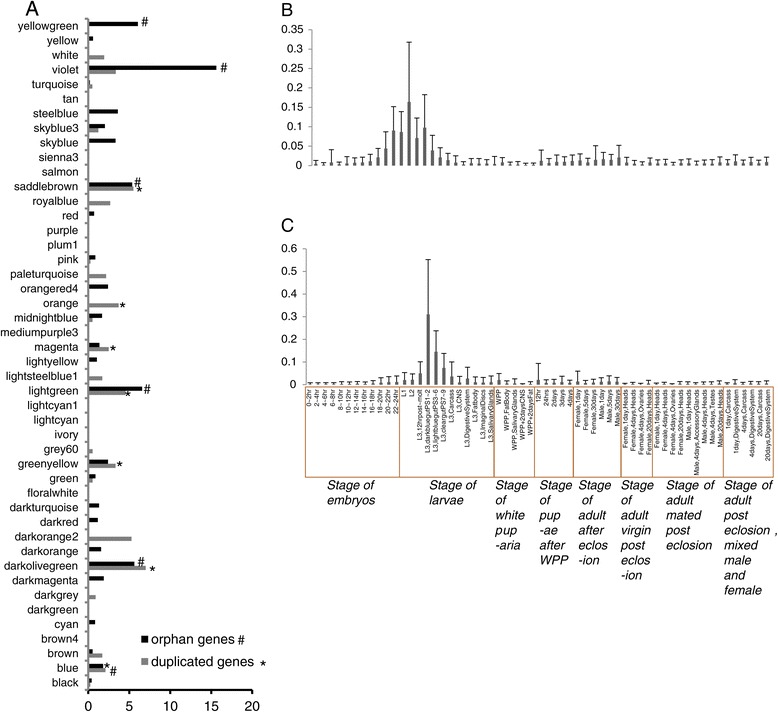
**Expression profile of new genes in each module. (A)** Enrichment of new genes (including duplicated genes and orphan genes) in each gene module. Different symbols are used to show modules with significant enrichment for duplicated and orphan genes. **(B-C)** Histogram of the expression of genes in modules M24 **(B)** and M37 **(C)** and heatmaps of the expression of genes in this modules are seeing in Additional files 9 and 10.

We identified a total of 221 new genes including newly duplicated and young orphan genes (detailed in Material and Methods), specific to *Drosophila melanogaster.* We then identified which modules, and developmental stages, were enriched with new genes in *Drosophila melanogaster*. Significant enrichment in new genes was found in 6 modules, M41, M24, M37, M13, M36 and M23 (P = 7.34E-14, P = 1.56E-11, P = 3.52E-09, P = 5.56E-08, P = 8.18E-07, and P = 2.39E-04, respectively, by the *χ*^2^ test, after FDR correction) (Additional file 8), where these modules are significantly correlated with WPP + 2 days Fat (P = 9e-37), stage L1 larvae (P = 1e-06, Figure 2B, Additional file 9), dark blue gut PS(1-2) in stage L3 larvae (P = 3e-15, Figure 2C, Additional file 10), 4 day post-eclosion testes in adult males (P = 3e-15), 12 hr post-molt stage L3 larvae (P = 4e-27) and 20-22 hr embryo stages (P = 1e-05), respectively (Additional file 3). Gene expression analysis of the new genes enriched in these six modules also showed consistent specific high levels of expression in their associated developmental stages, supporting potential roles of these new genes in these tissues/developmental stages (Additional file 11). PCA analysis (Principal Component Analysis) also found an inseparable relationship of the new genes with other genes in these modules, supporting the potential gene co-expression network (Additional file 12).

When we considered young duplicated genes, we found that young duplicated genes are significantly enriched in 7 modules, M37, M13, M24, M36, M23, M12 and M26 (P = 2.91E-06, P = 7.70E-06, P = 5.17E-05, P = 3.04E-04, P = 0.0013, P = 0.030 and P = 0.031, respectively, by the *χ*^2^ test, after FDR correction) (Figure 2A) (Additional file 13), which are significantly correlated with the dark blue gut PS(1-2) in stage L3 larvae (P = 3e-15, Figure 2C, Additional file 10), 4 day post-eclosion testes in adult males (P = 3e-15), stage L1 larvae (P = 1e-06, Figure 2B, Additional file 9), 12 hr post-molt stage L3 larvae (P = 4e-27), 20-22 hr embryos (P = 1e-05), 4 days post-eclosion accessory glands in adult males (P = 4E-32), and 18-20 hr embryo stages (P = 2e-17), respectively (Additional file 3). These observations support the previously reported testis-bias in the expression of some new genes, and that the function of these new genes in the evolution of phenotypes is determined by their expression in specific developmental stages. However, since as more tissues (59 development stages and tissues) and unique highly expressed genes were taken into account, only 40 genes among the 137 young genes are enriched in the M13 module correlated significantly with the testes. In addition, the significant clustering of the young genes with different co-expression modules that correlate with different tissues/developmental stages also suggests that the young genes might operate in many different functional classes, and not only in the testis. For example, gene ontology analysis of these 5 modules with young genes revealed that genes in the module M23 are enriched in functions that are integral component of membranes (intrinsic to membrane) (P = 0.0038, after FDR correction) and that genes in the module M24 are enriched in the aromatic amino acid family metabolic process (P = 0.035, after FDR correction) and the oxidation reduction process (P = 2.24E-07, after FDR correction), revealing potential functions for these new genes in these processes (Additional file 14).

Our searches identified 84 young orphan genes that show no homology with proteins in other species (and were not duplicated in the *D. melanogaster* lineage). 83 orphan genes were significantly enriched in 6 modules, M13, M37, M24, M36, M41 and M40 (P = 0.021, P = 0.0077, P = 3.02E-07, P = 0.0096, P = 2.88E-22 and P = 0.0052, respectively, by the *χ*^2^ test, after FDR correction) (Additional file 15, Figure 2A). Among these six modules, M13 is correlated with 4 day post-eclosion testes in adult males (P = 3e-15), M37 is correlated with dark blue gut PS(1-2) in stage L3 larvae (P = 3e-15, Figure 2C, Additional file 10), M24 is correlated with stage L1 larvae (P = 1e-06, Figure 2B, Additional file 9), M36 was correlated with 12 hr post-molt stage L3 larvae (P = 4e-27), M41 is corrected with WPP + 2 days Fat (P = 9e-37), and M40 is corrected with Mixed Adult Male and Female Carcass 4 days Post-eclosion (P = 2e-06) (Additional file 3), suggesting that these new genes have roles in these stages of development.

The young genes were then queried against the Bristle Screen online database [30] where we found that among the 111 young duplicated genes (from 181 lines) having knock-down data, 4 genes (from 6 lines) were completely lethal (Additional file 13). The proportion of young genes showing lethality (3.3%) was slightly lower than the genome wide value (8.9%, http://bristlescreen.imba.oeaw.ac.at/data_summary.php). Among the orphan genes, 52 (from 84 lines) have knock-down data in the Bristle Screen database, with only 1 gene (from 1 line) being completely lethal (Additional file 15). Our results, of a low proportion of lethality in young genes, contrasts with a previous conclusion that young genes have a similar proportion of lethality as old genes [21], a result that might have been attributable to the off target effects of RNAi on duplicated genes, producing false positives for the essentiality of the young duplicated genes. More accurate experimental methods for knocking down/out genes in *Drosophila* are necessary to evaluate the essentiality of young genes.

### Roles for specific lncRNAs in development revealed from the gene co-expression network analysis

**Figure 3:**
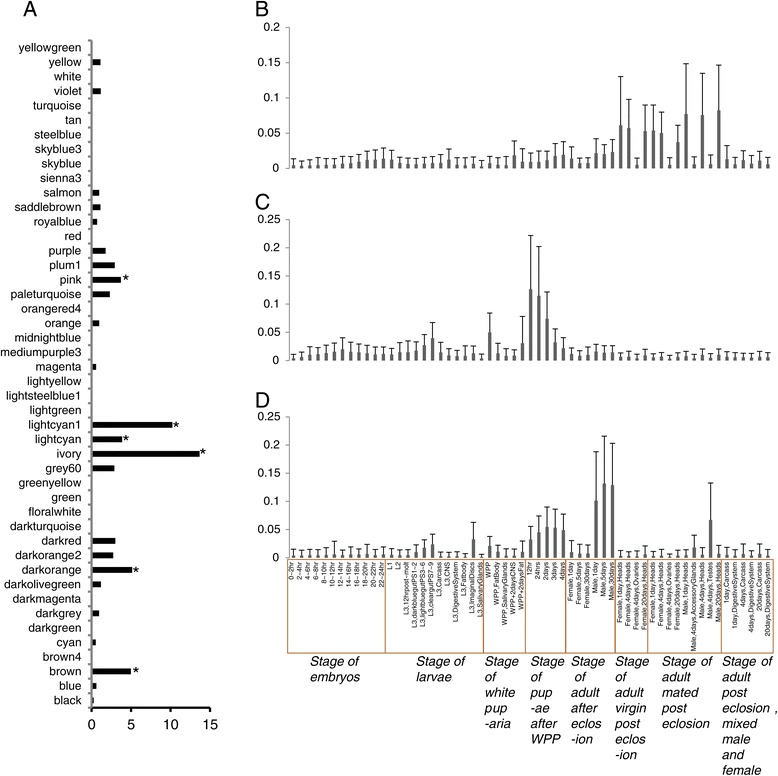
**Expression profile of lncRNAs in each module. (A)** Enrichment of lncRNAs in each gene module. Stars show modules with significant enrichment in lncRNAs. **(B-D)** Histogram of the expression of genes in modules M46 **(B)**, M20 **(C)** and M14 **(D)** and heatmaps of the expression of genes in this modules are seeing in Additional files 18, 19 and 20.

We assembled 18,385-26,178 new transcripts from the transcriptome data from 7 *Drosophila* species (Additional file 16). In *Drosophila melanogaster*, 145 of the newly assembled transcripts, from 133 genes, were identified as linage-specific lncRNAs that have no homology with transcripts in any other species. Among these new lncRNA genes, 129 were found to be significantly associated with 25 modules, with 6 of the modules, M14, M20, M42, M18, M3, and M46, significantly enriched with *Drosophila melanogaster* specific lncRNAs (P = 6.10E-31, P = 4.57E-04, P = 8.15E-12, P = 8.75E-04, P = 3.25E-07 and P = 3.82E-06, respectively, by the *χ*^2^ test, after FDR correction) (Figure 3A, Additional file 17). Of these six modules, M14 is correlated with the adult male 30 days after eclosion stage (Figure 3D, Additional file 18), M20 was correlated with the pupae 12 hr after WPP (P = 1e-06, Figure 3C, s Additional file 19), M18 is correlated with pupae 2 days after WPP (P = 3e-24), M42 is corrected with stage L3 CNS (P = 9e-20), M3 is corrected with stage L3 Imaginal Discs (P = 3e-28), and M46 is corrected with heads of mated adult males 20 days post-eclosion (P = 8e-04, Figure 3B, Additional files 3 and 20). We then used the genes in each of the modules where lncRNAs are enriched to conduct a gene ontology analysis, which may suggest potential functions for the lncRNAs. For example, genes in module M14 are enriched in odorant binding (P = 2.98e-24, after FDR correction), sensory perception of smell (P = 1.03E-17, after FDR correction), olfactory receptor activity (P = 7.73E-15, after FDR correction) and neurological system process (P = 3.57E-06, after FDR correction), suggesting a potential function of some of the lncRNAs in regulating expression of sensory perception genes. In addition, genes in module M20 are enriched in the defense response (P = 0.047, after FDR correction), genes in module M18 are enriched in proteolysis (P = 0.035, after FDR correction), and genes in module M46 are enriched in photo transduction (P = 1.06e-23, after FDR correction) (Additional file 21), suggesting that lncRNAs may have regulatory roles in these processes. Although the functions of the lncRNAs remain unclear, co-expression data provides information that could be examined in future studies.

### Essentiality of positively selected genes in *Drosophila*

9,746 one-to-one orthologs among *Drosophila melanogaster, D. annanassae, D. sechellia, D. yakuba and D. erecta* were retrieved from the EnsemblMetazoa database (http://metazoa.ensembl.org). After alignment and trimming, 9,531 orthologs remained for use in the detection of positive selection employing the branch site model of PAML [32]. A total of 263 and 383 Positive Selected Genes (PSGs) were identified in the *Drosophila melanogaster* and *D. sechellia* lineages, respectively. Of the one-to-one orthologs, 7,939 (from 13,784 lines) have knock-down data in the Bristle Screen database, with 929 (~11%) genes (from 1,291 lines) being completely lethal. Among the 263 PSGs, 216 genes (from 413 lines) with knock-down data in *Drosophila melanogaster*, with 36 of these genes (from 54 lines) being completely lethal (~13.08%) (Additional file 22). In *Drosophila sechellia*, among the 383 PSGs, 1 gene did not have detectable expression in the *Drosophila melanogaster* transcriptome datasets and 1 gene was placed in the non-sense module. The remaining 381 genes were distributed into 38 modules, with 40 (from 49 lines) of the 304 PSGs (from 553 lines) being completely lethal (Additional file 23).

Excluding one gene that had no detectable expression in the transcriptome datasets, 262 PSGs in *Drosophila melanogaster* were distributed into 36 modules and were significantly enriched in M6 (P = 0.002196, by the *χ*^2^ test, after FDR correction) (Additional file 22). Module M6 is significantly correlated with the 0-2 hr embryo stage (P = 4e-04), and this module is also significantly enriched with lethal genes (Figure 1C, Additional file 5). Enrichment of lethal genes also points to the importance of module M6. Among the 36 PSGs with completely lethality in *Drosophila melanogaster,* 18 are distributed to module M6. These data demonstrate that PSGs have essential functions, although they show rapid evolution with amino acid sequence changes driven by positive selection.

## Discussion

### Roles for new genes in development revealed by gene co-expression network analysis

Early developmental stages are very important for establishing phenotypes of later stages. Based on our gene co-expression network analysis, we observed that the non-random distribution of essential genes at early developmental stages in *Drosophila* development leads to different developmental stages showing varying levels of essentiality. For example, the 0-2 hr embryonic stage, which is the maternal to zygotic transition period in *Drosophila*, has enriched expression of essential genes, suggesting that the contribution of gene expression of these genes in this period of developmental time is important for phenotype evolution in *Drosophila*. Similarly, we found that the expression of new genes, including young protein coding and lncRNA genes, are also not randomly distributed during early development, and is significantly enriched in modules that correlate with specific developmental stages. This also suggests that these new genes also play roles in the evolution of specific phenotypes. New genes provide crucial material for evolutionary innovation, and evidence has been accumulating supporting the remarkable phenotypic effects of new genes in development. A pioneering study by Chen et al (2010) found that many new genes rapidly evolve essential functions in *Drosophila*, as the knockdown of expression many of these new genes by RNA interference (RNAi) leads to lethality at various stages of *Drosophila* development [21]. Enrichment of new genes in early developmental stages supports a role for adaptive function by new genes at early developmental stages. For example, in human, the recruitment of young genes into the early developmental stages of neocortex was found to account potentially for the evolution of cognitive ability [23]. Many studies have found a remarkable divergence between related species both in their early and late development, which is consistent with the hourglass model of development, although it has been proposed by others that genes expressed in early stages of development should be highly conserved [33]. Recruitment of newly originated genes at early stages of developmental might allow evolution of specific phenotypes, while conserved genes retain function for the conserved core of development.

### Roles for positively selected genes in development

With the advent of large-scale genomic data, large numbers of positively selected adaptive mutations in the coding sequences of genes involved in the evolution of phenotypes, including morphological and physiological traits, have been identified [14]. Here, positively selected genes showing rapid amino acid sequence change specific to the *Drosophila melanogaster* lineage were identified, with some of these genes found to be essential as knock-down leads to lethality. The correlation between evolutionary rate and essentiality of genes is weak [34], where gene essentiality tends to be associated with adaptive evolution in protein sequence. Although, the functional shifts caused by amino acid changes in PSGs are often unclear, mutations driven by positive selection appear to contribute significantly to the evolution of the phenotypes generated by the PSGs. Enrichment of PSGs among gene co-expression modules correlated with early developmental stages supports the hypothesis that changes in the amino acid sequences of PSGs leads to phenotypic evolution.

## Conclusions

Young genes, including duplicated, orphan, and young lncRNA genes, are significantly enriched among modules associated with specific developmental stages. In addition, genes undergoing rapid amino acid sequence evolution driven by positive selection showed a similar proportion of essentiality with other genes, and enrichment at module of specific developmental stages. Our integrative analysis revealed important roles for the origin of new genes and rapid amino acid sequence evolution in development that may account for specific phenotype evolution in *Drosophila melanogaster* to some extent.

## Methods

### RNA-seq transcriptome data for *Drosophila melanogaster* and other *Drosophila* species

Transcriptome data for *Drosophila melanogaster*, including 27 distinct developmental stages and 12 tissues from different development stages [26], and data from six other *Drosophila* species (*D. ananassae, D. mojavensis, D. pseudoobscura, D. simulans, D. virilis,* and *D. yakuba*) were downloaded from the SRA database (http://www.ncbi.nlm.nih.gov/sra/) (Additional file 1).

### Analysis of transcript expression with Tophat and Cufflinks

Transcriptome raw read data, in sra format, was converted to fastq using the SRA Toolkit (available at http://www.ncbi.nlm.nih.gov/Traces/sra/?view=software), and trimmed with Btrim [35]. Tophat [36] (v2.0.4) was implemented to align the reads, by default, and Cufflinks (v 2.0.2) was used to assemble transcripts with the –g parameter. Cuffcompare was used to generate an integrated combined gtf annotation file along with the Ensembl annotation file. New transcripts with class codes “c, j, e, i, o, u, x”, having more than 2 exons, lengths longer than 200 bp, and expression levels from FPKM values higher than 1.0 in at least one sample, were retrieved, and merged with the reference annotated gtf file. The merged gtf file was used as input for Cufflinks to calculate expression values for each gene with the –G parameter.

### Detection of lncRNAs specific to *Drosophila melanogaster*

The coding potential of transcripts from the newly assembled loci was evaluated by the coding Potential Calculator (CPC) [37], and values lower than -1 were considered to be noncoding. All transcripts in the other *Drosophila* species (*D. ananassae, D. mojavensis, D. pseudoobscura, D. simulans, D. virilis,* and *D. yakuba*), including annotated cDNA and newly assembled transcripts, were merged. *Drosophila melanogaster* lncRNAs were used as the queries for BLASTN searches against the merged cDNA with a cutoff e-value 1e-10 [38]. *Drosophila melanogaster* lncRNAs without homologous hits in the other species were treated as *Drosophila melanogaster* specific.

### WGCNA (weighted gene co-expression network analysis)

WGCNA (weighted gene co-expression network analysis) package implemented in R [27] was used to construct a weighted gene network based on the expression values calculated above. The power of 8, for which the scale-free topology fit index curves flatten out at roughly 0.9, were interpreted as a soft-threshold for the adjacency matrix. In total, 46 modules were identified. Unassigned genes, 37genes, were placed into the “grey” module (module M47). Module membership, defined as the Pearson correlation between the expression level of a given gene and a given module eigengene was calculated to identify intramodular hub genes. Modules whose eigengenes (referred to as the first principal component) were highly correlated with different biological stages were merged.

Each module was summarized by a single representative expression profile, which was referred to as the module eigengene. The module eigengene is a more complete representation of gene co-expression relationship in each network. We correlated eigengenes with various developmental stages to access the association between a module and a developmental stage. For example, a correlation, like 0.97, indicates a high association between one gene co-expression network and one developmental stage.

### Identification of new genes in *Drosophila melanogaster*

Ages of genes in *Drosophila melanogaster* were retrieved from ProteinHistorian [39]. ProteinHistorian estimated gene ages by making use of different ancestral family reconstruction algorithms and pre-existing protein family database and phylogenetic trees. Duplicated genes in *Drosophila melanogaster* were retrieved from Ensembl using Biomart [40]. However, Ensembl database dates when the duplication occurred, but does not provide information that which gene is the source copy and which is the derived copy. Here, we defined young duplicated genes (derived copies) as these duplicated genes with an age of 0 induced by ProteinHistorian. Protein sequences in *Drosophila melanogaster* were used to BLASTP against the protein database of other *Drosophila* with an e-value 1e-10 [38] (1e-3 and 1e-4 were used and the results, seeing in Additional files 24 and 25, were very similar with 1e-10). The genes in *Drosophila melanogaster* having no homologous hits in the other species were treated as orphan genes.

### Detection of positive selection in protein coding genes

Orthologous_one2one genes among melanogaster group: *Drosophila sechellia, D. menlanogaster, D. yakuba, D. erecta,* and *D. ananassae* were retrieved using Biomart from EnsemblMetazoa (http://metazoa.ensembl.org). PRANK (available at http://www.ebi.ac.uk/goldman-srv/prank/prank/) was used for multiple sequence alignments at the codon level. To obtain high quality sequence alignments, the longest transcript of each species was chosen when genes had more than one transcript and sequencing errors and non-orthologous sequences were removed using an in-house script. Only alignments with lengths longer than 30 codons were retained. The accepted phylogenetic relationship of the melanogaster group was used (((*Drosophila schellia, D. melanogaster*), (*D. yakuba*, *D. erecta*)), *D. ananassae*). The CODEML algorithm from the PAML4 package [41] was used to detect positive selection utilizing the branch-site model [32].

### Gene ontology analysis

The Database for Annotation visualization and Integrated Discovery (DAVID) was used to investigate the enrichment of Gene Ontology (GO) terms and KEGG pathways [42]. In order to avoid a selection bias, we also performed the GO analysis using the goseq [43] package (http://www.bioconductor.org/packages/release/bioc/html/goseq.html) in R (v-2.15.3). The analysis was corrected for the length of *Drosophila melanogaster* reference genes. FDR correction was performed using the package “multtest” (available in http://www.bioconductor.org/packages/release/bioc/html/multtest.html) with the parameter “proc = BH”.

## Additional files

Additional file 1:
**Transcriptome data used in this study.**
Additional file 2:
**Coexpression modules identified in this study labeled numerically and in color.**
Additional file 3:
**Table presenting the correlations (and corresponding p-values) between modules and developmental stages and adult tissues.**
Additional file 4:
**Heatmap of the expression of genes in module M17, expression of genes in which are significantly associated with the white prepupae (WPP) stages.**
Additional file 5:
**Heatmap of the expression of genes in module M6, expression of genes in which are significantly associated with the 0-2 hr embryo stage.**
Additional file 6:
**Heatmap of the expression of genes in mouse M28, expression of genes in which are significantly associated with the 10-12 hr embryo development stage.**
Additional file 7:
**Gene ontology analysis of genes in the M6 module, with the results from DAVID and GOseq.**
Additional file 8:
**New genes, with the results from the Bristle Screen online database and membership in the modules of co-expressed genes.**
Additional file 9:
**Heatmap of the expression of genes in module M24, expression of genes in which are significantly associated with the stage L1 larvae.**
Additional file 10:
**Heatmap of the expression of genes in module M37, expression of genes in which are significantly associated with the dark blue gut PS(1-2) in stage L3 larvae.**
Additional file 11:
**Histogram of the expression of new genes in modules M13 (A), M37 (B), M23 (C), M24 (D), M36 (E) and M41 (F).**
Additional file 12:
**The PCA (Principal Component Analysis) of modules M13 (A), M37 (B), M23 (C), M24 (D), M36 (E) and M41 (F).**
Additional file 13:
**Newly duplicated genes, with results from the Bristle Screen online database and membership in modules of co-expressed genes.**
Additional file 14:
**Gene ontology analysis of genes in the M23 and M24 modules, with results from DAVID and GOseq.**
Additional file 15:
**Young orphan genes (BLASTP with e-value 1e-10), with the results from the Bristle Screen online database and membership in the modules of co-expressed genes.**
Additional file 16:
**Total and newly assembled transcripts from 7** ***Drosophila*** **species.**
Additional file 17:
**Detection of lncRNAs we detected and membership in the modules of co-expressed genes.**
Additional file 18:
**Heatmap of the expression of genes in module M14, expression of genes in which are significantly associated with the adult male 30 days after eclosion stage.**
Additional file 19:
**Heatmap of the expression of genes in module M20, expression of genes in which are significantly associated with the pupae 12 hr after WPP.**
Additional file 20:
**Heatmap of the expression of genes in module M46, expression of genes in which are significantly associated with the heads of mated adult males 20 days post-eclosion.**
Additional file 21:
**Gene ontology analysis of genes in the M14, M20, M18, M41 and M45 modules, with results from DAVID and GOseq.**
Additional file 22:
**PSGs in** ***Drosophila melanogaster*** **, with the results from the Bristle Screen online database and membership in the modules of co-expressed genes.**
Additional file 23:
**PSGs in** ***Drosophila sechellia*** **, with the results from the Bristle Screen online database and membership in the modules of co-expressed genes.**
Additional file 24:
**Young orphan genes (BLASTP with e-value 1e-3), with the results from the Bristle Screen online database and membership in the modules of co-expressed genes.**
Additional file 25:
**Young orphan genes (BLASTP with e-value 1e-4), with the results from the Bristle Screen online database and membership in the modules of co-expressed genes.**

## References

[1] Britten RJ, Davidson EH (1971). Repetitive and non-repetitive DNA sequences and a speculation on the origins of evolutionary novelty. Q Rev Biol.

[2] King M-C, Wilson AC (1975). Evolution at two levels in humans and chimpanzees. Science.

[3] Carroll SB (2007). The Making of the Fittest: DNA and the Ultimate Forensic Record of Evolution.

[4] Carroll SB (2005). Evolution at two levels: on genes and form. PLoS Biol.

[5] Carroll SB (2005). Endless Forms Most Beautiful: The New Science of Evo Devo and the Making of the Animal Kingdom.

[6] Carroll SB (2008). Evo-devo and an expanding evolutionary synthesis: a genetic theory of morphological evolution. Cell.

[7] Enard W, Gehre S, Hammerschmidt K, Hölter SM, Blass T, Somel M, Brückner MK, Schreiweis C, Winter C, Sohr R, Becker L, Wiebe V, Nickel B, Giger T, Müller U, Groszer M, Adler T, Aguilar A, Bolle I, Calzada-Wack J, Dalke C, Ehrhardt N, Favor J, Fuchs H, Gailus-Durner V, Hans W, Hölzlwimmer G, Javaheri A, Kalaydjiev S, Kallnik M (2009). A humanized version of Foxp2 affects cortico-basal ganglia circuits in mice. Cell.

[8] Zhang J (2003). Evolution of the human ASPM gene, a major determinant of brain size. Genetics.

[9] Vallender EJ, Lahn BT (2004). Positive selection on the human genome. Hum Mol Genet.

[10] Evans PD, Anderson JR, Vallender EJ, Gilbert SL, Malcom CM, Dorus S, Lahn BT (2004). Adaptive evolution of ASPM, a major determinant of cerebral cortical size in humans. Hum Mol Genet.

[11] Evans PD, Anderson JR, Vallender EJ, Choi SS, Lahn BT (2004). Reconstructing the evolutionary history of microcephalin, a gene controlling human brain size. Hum Mol Genet.

[12] Y-q W, Su B (2004). Molecular evolution of microcephalin, a gene determining human brain size. Hum Mol Genet.

[13] Enard W, Przeworski M, Fisher SE, Lai CSL, Wiebe V, Kitano T, Monaco AP, Paabo S (2002). Molecular evolution of FOXP2, a gene involved in speech and language. Nature.

[14] Hoekstra HE, Coyne JA (2007). The locus of evolution: evo devo and the genetics of adaptation. Evolution.

[15] Boyko AR, Williamson SH, Indap AR, Degenhardt JD, Hernandez RD, Lohmueller KE, Adams MD, Schmidt S, Sninsky JJ, Sunyaev SR (2008). Assessing the evolutionary impact of amino acid mutations in the human genome. PLoS Genet.

[16] Gojobori J, Tang H, Akey JM, Wu C-I (2007). Adaptive evolution in humans revealed by the negative correlation between the polymorphism and fixation phases of evolution. Proc Natl Acad Sci U S A.

[17] Stern DL, Orgogozo V (2009). Is genetic evolution predictable?. Science.

[18] Wu D-D, Zhang Y-P (2013). Evolution and function of de novo originated genes. Mol Phylogenet Evol.

[19] Chen S, Krinsky BH, Long M (2013). New genes as drivers of phenotypic evolution. Nat Rev Genet.

[20] Long M, VanKuren NW, Chen S, Vibranovski MD (2013). New gene evolution: little did we know. Ann Rev Genet.

[21] Chen S, Zhang YE, Long M (2010). New genes in drosophila quickly become essential. Science.

[22] Wu D-D, Irwin DM, Zhang Y-P (2011). De novo origin of human protein-coding genes. PLoS Genet.

[23] Zhang YE, Landback P, Vibranovski MD, Long M (2011). Accelerated recruitment of new brain development genes into the human genome. PLoS Biol.

[24] Chen S, Spletter M, Ni X, White KP, Luo L, Long M (2012). Frequent recent origination of brain genes shaped the evolution of foraging behavior in *drosophila*. Cell Rep.

[25] Li C-Y, Zhang Y, Wang Z, Zhang Y, Cao C, Zhang P-W, Lu S-J, Li X-M, Yu Q, Zheng X, Du Q, Uhl GR, Liu Q-R, Wei L (2010). A human-specific de novo protein-coding gene associated with human brain functions. PLoS Comput Biol.

[26] Graveley BR, Brooks AN, Carlson JW, Duff MO, Landolin JM, Yang L, Artieri CG, Baren MJV, Boley N, Booth BW, Brown JB, Cherbas L, Davis CA, Dobin A, Li R, Lin W, Malone JH, Mattiuzzo NR, Miller D, Sturgill D, Tuch BB, Zaleski C, Zhang D, Blanchette M, Dudoit S, Eads B, Green RE, Hammonds A, Jiang L, Kapranov P (2011). The developmental transcriptome of drosophila melanogaster. Nature.

[27] Langfelder P, Horvath S (2008). WGCNA: an R package for weighted correlation network analysis. BMC Bioinformatics.

[28] Hawrylycz MJ, Lein ES, Guillozet-Bongaarts AL, Shen EH, Ng L, Miller JA, van de Lagemaat LN, Smith KA, Ebbert A, Riley ZL, Abajian C, Beckmann CF, Bernard A, Bertagnolli D, Boe AF, Cartagena PM, Chakravarty MM, Chapin M, Chong J, Dalley RA, Daly BD, Dang C, Datta S, Dee N, Dolbeare TA, Faber V, Feng D, Fowler DR, Goldy J, Gregor BW (2012). An anatomically comprehensive atlas of the adult human brain transcriptome. Nature.

[29] Kang HJ, Kawasawa YI, Cheng F, Zhu Y, Xu X, Li M, Sousa AMM, Pletikos M, Meyer KA, Sedmak G, Guennel T, Shin Y, Johnson MB, Krsnik Ž, Mayer S, Fertuzinhos S, Umlauf S, Lisgo SN, Vortmeyer A, Weinberger DR, Mane S, Hyde TM, Huttner A, Reimers M, Kleinman JE, Šestan N (2011). Spatio-temporal transcriptome of the human brain. Nature.

[30] Mummery-Widmer JL, Yamazaki M, Stoeger T, Novatchkova M, Bhalerao S, Chen D, Dietzl G, Dickson BJ, Knoblich JA (2009). Genome-wide analysis of Notch signalling in Drosophila by transgenic RNAi. Nature.

[31] Tadros W, Lipshitz HD (2009). The maternal-to-zygotic transition: a play in two acts. Development.

[32] Zhang J, Nielsen R, Yang Z (2005). Evaluation of an improved branch-site likelihood method for detecting positive selection at the molecular level. Mol Biol Evol.

[33] Nei M (2007). The new mutation theory of phenotypic evolution. Proc Natl Acad Sci U S A.

[34] Koonin EV: **Systemic determinants of gene evolution and function.** *Mol Syst Biol* 2005, **1**(1):ᅟ. Epub. 10.1038/msb4100029 PMC1681469 16729056

[35] Kong Y (2011). Btrim: a fast, lightweight adapter and quality trimming program for next-generation sequencing technologies. Genomics.

[36] Trapnell C, Pachter L, Salzberg SL (2009). TopHat: discovering splice junctions with RNA-Seq. Bioinformatics.

[37] Kong L, Zhang Y, Ye Z-Q, Liu X-Q, Zhao S-Q, Wei L, Gao G (2007). CPC: assess the protein-coding potential of transcripts using sequence features and support vector machine. Nucleic Acids Res.

[38] Altschul SF, Madden TL, Schaffer AA, Zhang J, Zhang Z, Miller W, Lipman DJ (1997). Gapped BLAST and PSI-BLAST: a new generation of protein database search programs. Nucleic Acids Res.

[39] Prlic A, Capra JA, Williams AG, Pollard KS (2012). ProteinHistorian: tools for the comparative analysis of eukaryote protein origin. PLoS Comput Biol.

[40] Smedley D, Haider S, Ballester B, Holland R, London D, Thorisson G, Kasprzyk A (2009). BioMart-biological queries made easy. BMC Genomics.

[41] Yang Z (2007). PAML 4: phylogenetic analysis by maximum likelihood. Mol Biol Evol.

[42] Dennis G, Sherman BT, Hosack DA, Yang J, Gao W, Lane HC, Lempicki RA (2003). DAVID: database for annotation, visualization, and integrated discovery. Genome Biol.

[43] Young MD, Wakefield MJ, Smyth GK, Oshlack A (2010). Gene ontology analysis for RNA-seq: accounting for selection bias. Genome Biol.

