# Supplementary material for: Integrative analysis of young genes, positively selected genes and lncRNAs in the development of Drosophila melanogaster
Source: BMC Evol Biol. 2014 Dec 4;14:241. doi: 10.1186/s12862-014-0241-9 (PMC4258281; doi:10.1186/s12862-014-0241-9)
Supplement: Additional file 4: — Heatmap of the expression of genes in module M17, expression of genes in which are significantly associated with the white prepupae (WPP) stages. [file 12862_2014_241_MOESM4_ESM.pdf]

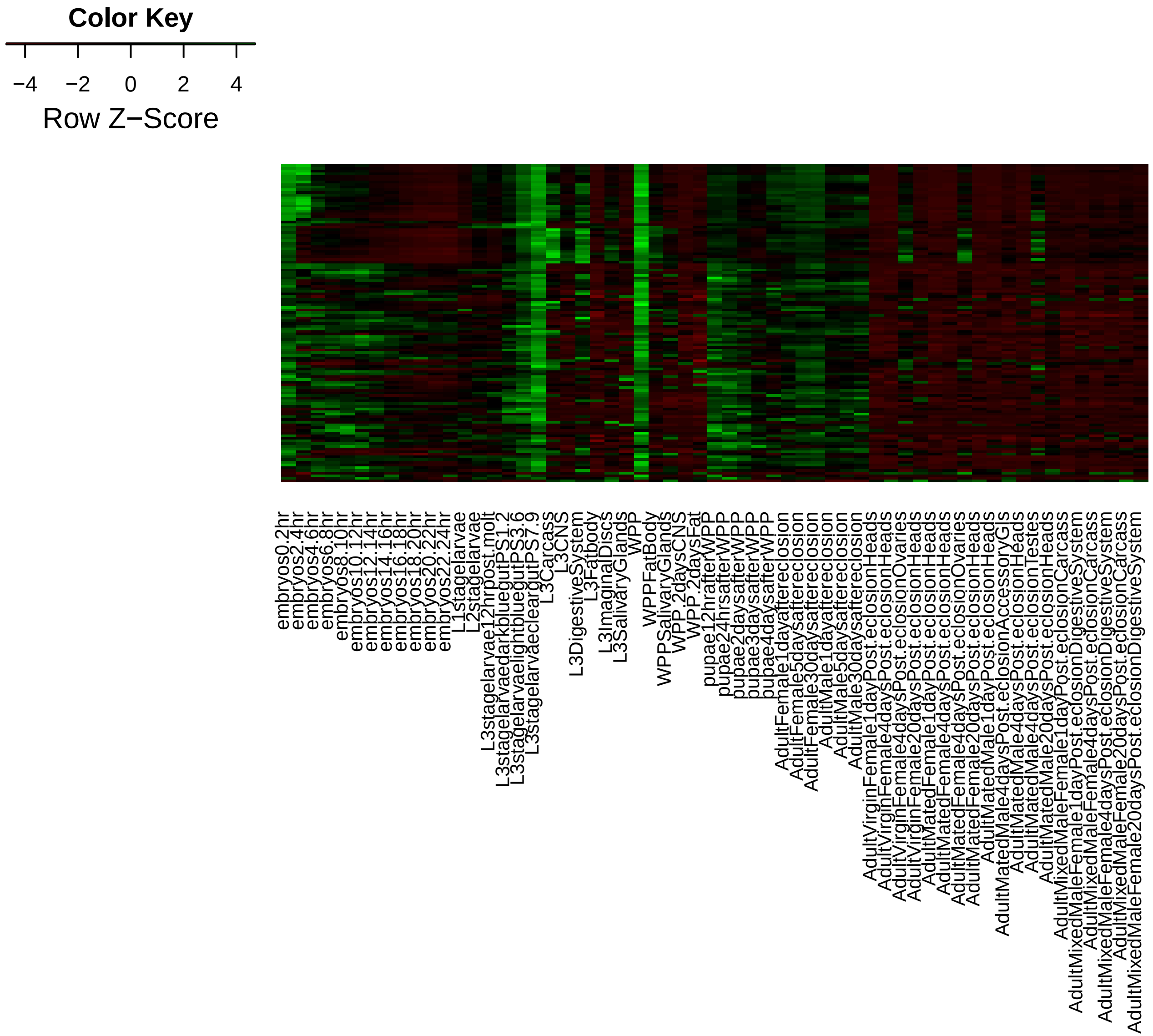

Color Key
−4
−2
0
2
4
Row Z−Score
embryos0.2hr
embryos2.4hr
embryos4.6hr
embryos6.8hr
embryos8.10hr
embryos10.12hr
embryos12.14hr
embryos14.16hr
embryos16.18hr
embryos18.20hr
embryos20.22hr
embryos22.24hr
L1stagelarvae
L2stagelarvae
L3stagelarvae12hrpost.molt
L3stagelarvaedarkbluegutPS1.2
L3stagelarvaelightbluegutPS3.6
L3stagelarvaecleargutPS7.9
L3Carcass
L3CNS
L3DigestiveSystem
L3Fatbody
L3ImaginalDiscs
L3SalivaryGlands
WPP
WPPFatBody
WPPSalivaryGlands
WPP.2daysCNS
WPP.2daysFat
pupae12hrafterWPP
pupae24hrsafterWPP
pupae2daysafterWPP
pupae3daysafterWPP
pupae4daysafterWPP
AdultFemale1dayaftereclosion
AdultFemale5daysaftereclosion
AdultFemale30daysaftereclosion
AdultMale1dayaftereclosion
AdultMale5daysaftereclosion
AdultMale30daysaftereclosion
AdultVirginFemale1dayPost.eclosionHeads
AdultVirginFemale4daysPost.eclosionHeads
AdultVirginFemale4daysPost.eclosionOvaries
AdultVirginFemale20daysPost.eclosionHeads
AdultMatedFemale1dayPost.eclosionHeads
AdultMatedFemale4daysPost.eclosionHeads
AdultMatedFemale4daysPost.eclosionOvaries
AdultMatedFemale20daysPost.eclosionHeads
AdultMatedMale1dayPost.eclosionHeads
AdultMatedMale4daysPost.eclosionAccessoryGls
AdultMatedMale4daysPost.eclosionHeads
AdultMatedMale4daysPost.eclosionTestes
AdultMatedMale20daysPost.eclosionHeads
AdultMixedMaleFemale1dayPost.eclosionCarcass
AdultMixedMaleFemale1dayPost.eclosionDigestiveSystem
AdultMixedMaleFemale4daysPost.eclosionCarcass
AdultMixedMaleFemale4daysPost.eclosionDigestiveSystem
AdultMixedMaleFemale20daysPost.eclosionCarcass
AdultMixedMaleFemale20daysPost.eclosionDigestiveSystem
