# Supplementary figures and images for: Integrative analysis of young genes, positively selected genes and lncRNAs in the development of Drosophila melanogaster
Source: BMC Evol Biol. 2014 Dec 4;14:241. doi: 10.1186/s12862-014-0241-9 (PMC4258281; doi:10.1186/s12862-014-0241-9)

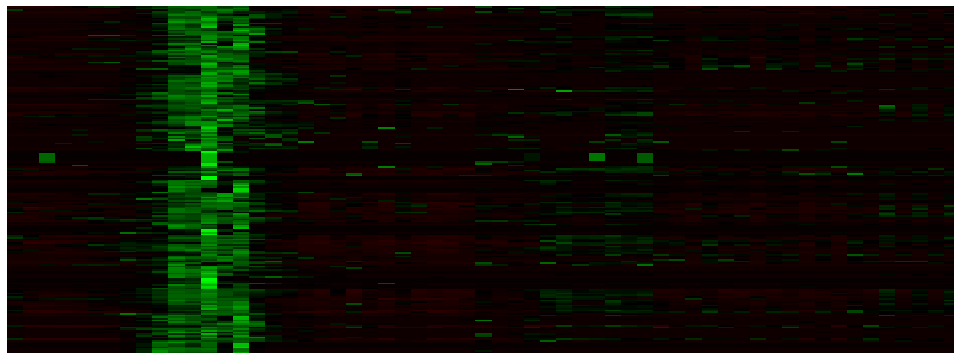[illegible]

Supplement: Additional file 9: — Heatmap of the expression of genes in module M24, expression of genes in which are significantly associated with the stage L1 larvae. [file 12862_2014_241_MOESM9_ESM.pdf]

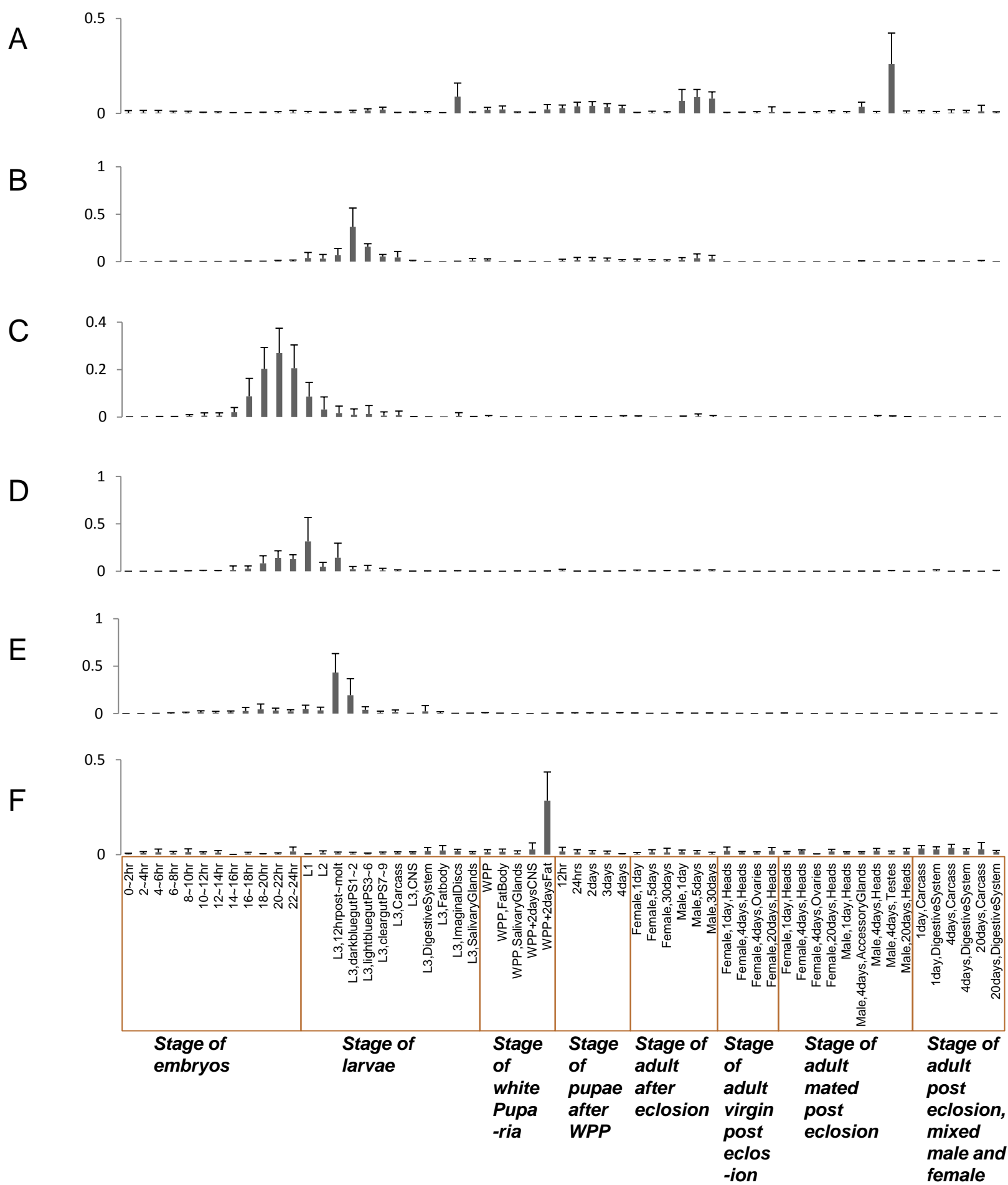

Supplement: Additional file 11: — Histogram of the expression of new genes in modules M13 (A), M37 (B), M23 (C), M24 (D), M36 (E) and M41 (F). [file 12862_2014_241_MOESM11_ESM.pdf]

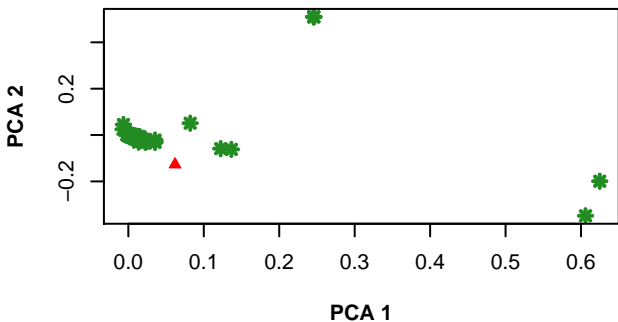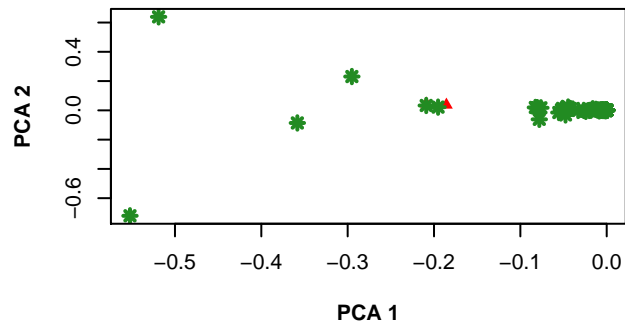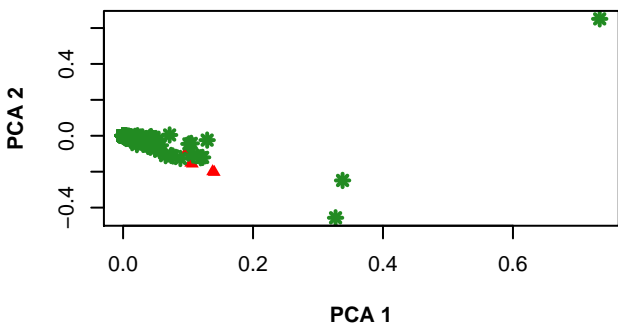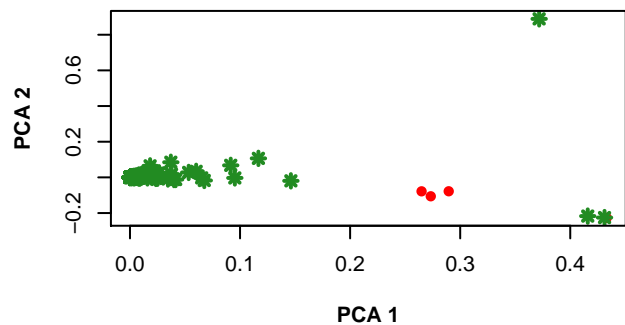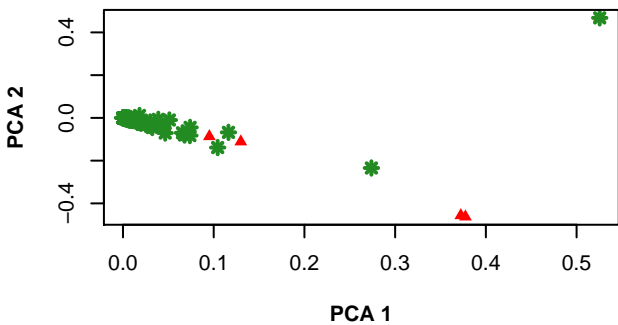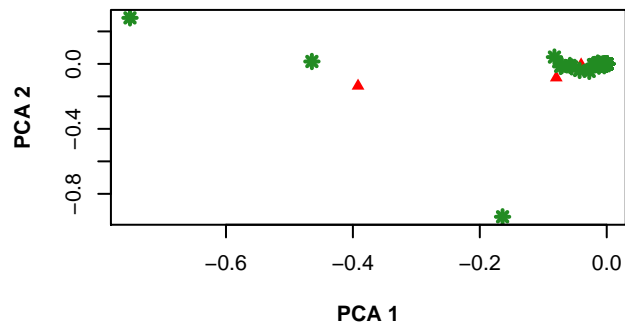

Supplement: Additional file 12: — The PCA (Principal Component Analysis) of modules M13 (A), M37 (B), M23 (C), M24 (D), M36 (E) and M41 (F). [file 12862_2014_241_MOESM12_ESM.pdf]

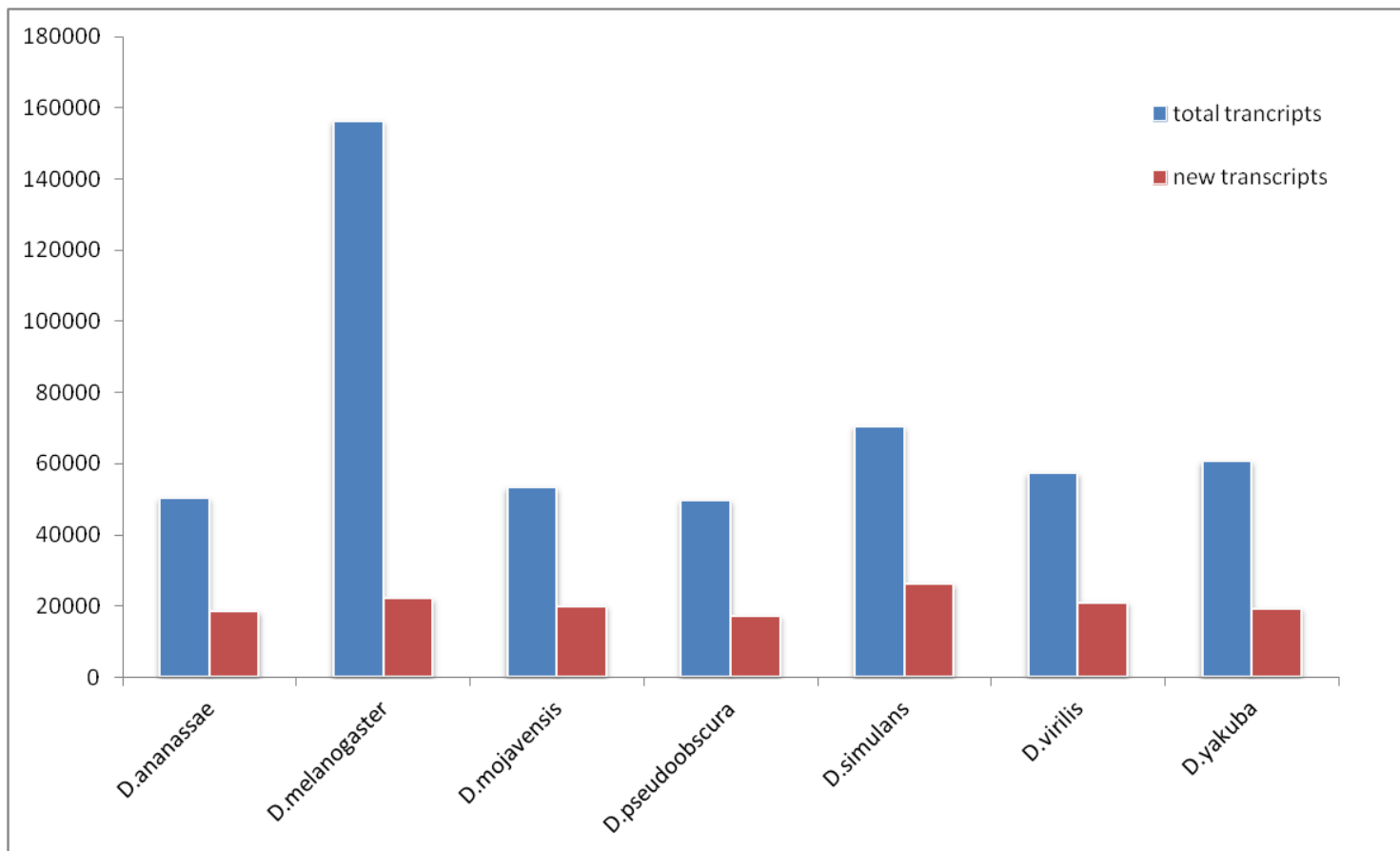

Supplement: Additional file 16: — Total and newly assembled transcripts from 7 Drosophila species. [file 12862_2014_241_MOESM16_ESM.pdf]
